# Extreme loss of immunoreactive p-Akt and p-Erk1/2 during routine fixation of primary breast cancer

**DOI:** 10.1186/bcr2719

**Published:** 2010-09-28

**Authors:** Isabel F Pinhel, Fiona A MacNeill, Margaret J Hills, Janine Salter, Simone Detre, Roger A'Hern, Ashutosh Nerurkar, Peter Osin, Ian E Smith, Mitch Dowsett

**Affiliations:** 1 Department of Academic Biochemistry, Institute of Cancer Research, Fulham Road, London, SW3 6JJ, UK; 2 Breast Unit, Royal Marsden Hospital, Fulham Road, London, SW3 6JJ, UK; 3 Department of Academic Biochemistry, Royal Marsden Hospital, Fulham Road, London, SW3 6JJ, UK; 4 Breakthrough Breast Cancer Research Centre, Institute of Cancer Research, Fulham Road, London, SW3 6JB, UK; 5 Clinical Trials and Statistics Unit, Institute of Cancer Research, Cotswold Road, Sutton, SM2 5NG, UK; 6 Department of Histopathology, Royal Marsden Hospital, Fulham Road, London, SW3 6JJ, UK

## Abstract

**Introduction:**

Very few studies have investigated whether the time elapsed between surgical resection and tissue fixation or the difference between core-cut and excision biopsies impact on immunohistochemically measured biomarkers, including phosphorylated proteins in primary breast cancer. The aim of this study was to characterise the differences in immunoreactivity of common biomarkers that may occur (1) as a result of tissue handling at surgery and (2) between core-cuts and resected tumours.

**Methods:**

Core-cuts taken from surgical breast cancer specimens immediately after resection (sample A) and after routine X-ray of the excised tumour (sample B) were formalin-fixed and paraffin-embedded and compared with the routinely fixed resection specimen (sample C). The variation in immunohistochemical expression of Ki67, oestrogen receptor (ER), progesterone receptor (PgR), human epidermal growth factor 2 (HER2), p-Akt and p-Erk1/2 were investigated.

**Results:**

Twenty-one tissue sets with adequate tumour were available. Median time between collection of core-cuts A and B was 30 minutes (range, 20 to 80 minutes). None of the markers showed significant differences between samples A and B. Similarly, Ki67, ER, PgR and HER2 did not differ significantly between core-cuts and main resection specimen, although there was a trend for lower resection values for ER (*P *= 0.06). However, p-Akt and p-Erk1/2 were markedly lower in resections than core-cuts (median, 27 versus 101 and 69 versus 193, respectively; both *P *< 0.0001 [two-sided]). This difference was significantly greater in mastectomy than in lumpectomy specimens for p-Erk1/2 (*P *= 0.01).

**Conclusions:**

The delay in fixation in core-cuts taken after postoperative X-ray of resection specimens has no significant impact on expression of Ki67, ER, PgR, HER2, p-Akt or p-Erk1/2. However, extreme loss of phospho-staining can occur during routine fixation of resection specimens. These differences are likely attributable to suboptimal fixation and may have major repercussions for clinical research involving these markers.

## Introduction

Stratification of therapy is a prime goal of current research. Tissue biomarkers are expected to provide indices enabling selection of therapy. Assurance of the validity of biomarker measurement is critical for their accurate application and interpretation, particularly in the context of presurgical studies, which are being increasingly used to speed drug development [1].

A variety of tissue sample types (e.g., core-cuts, punch biopsies, excisions) are used in biomarker studies, and comparative measurements of a marker between tissue types may occur within a single trial (e.g., core-cut at diagnosis/pretreatment versus excision/posttreatment). There are, however, few data available on the impact of sample type, even for frequently measured biomarkers such as Ki67, which has been used as a primary end point of some trials [2]. It is essential that any differences that arise from the expression of such markers in trials should be solely attributable to the effect of treatment with the drug and not due to potential artefacts such as those caused by delays to tissue fixation in different sample types.

Protein kinases are targets for approximately one third of drugs in development for oncology. Their phosphorylated products provide pharmacodynamic end points during clinical development and are likely, at least in some cases, to be determinants/indices of treatment efficacy and thus to become biomarkers in routine practice. Some previous studies have indicated that some phosphoproteins are labile during fixation [3,4]. We undertook a systematic assessment of immunoreactive expression of several established or developmental biomarkers for breast cancer, including two centrally important phosphorylated proteins: p-Akt and p-Erk1/2. To address these issues, we evaluated two situations that arise in the increasingly popular "window of opportunity" studies in primary breast cancer that exploit the approximately 2 weeks between diagnosis and surgery: (1) the delay of starting fixation between tumour excision and its return to theatre after X-ray to assess calcification and margin clearance, and (2) differences between core-cuts fixed immediately on tumour resection and histopathological sections from routinely fixed primary breast cancers.

## Materials and methods

### Sample collection

Twenty-eight patients were studied at resection of primary breast cancer; 29 specimens were available (one patient with two tumours). Basic demographics were median age, 54 years; median tumour size, 29 mm; lumpectomy versus mastectomy, 16 versus 13, respectively; node negative versus positive, 16 and 12, respectively (one was not available). Two 14-gauge core-cuts were taken immediately after tumour resection (sample A); one was placed in neutral-buffered formalin and one into RNAlater (Applied Biosystems/Ambion, Austin, TX, USA). The tumour was sent for X-ray at ambient temperature; on return to theatre after a recorded time, two more core-cuts were taken and handled similarly (sample B). The resected specimen (sample C) was placed in formalin and subjected to the histopathology department's routine fixation for breast tumours: lumpectomy specimens were left unsliced until the next morning, and mastectomy specimens were sliced at intervals of about 10 mm to allow penetration of formalin. Radioactive specimens were left unsliced for 48 hours to allow for isotope decay. Ethical approval was provided by the Royal Marsden Hospital (RMH). All patients gave written, informed consent.

### Immunohistochemical determination of Ki67, ER, PgR, HER2, p-Akt and p-Erk1/2

Four-micron sections of formalin-fixed, paraffin-embedded (FFPE) tissues were deparaffinised in xylene for 5 minutes and gradually rehydrated in decreasing grades (100%, 90%, 80% and 70%) of industrial methylated spirits (IMS) and water washes. Table 1 provides further details of the immunohistochemical methodology used for all markers. In all cases, endogenous peroxidase blocking, incubation with primary and secondary antibodies and signal amplification and detection steps of the immunohistochemical procedure were conducted on the Autostainer Immunostaining System (Dako, Carpinteria, CA, USA). Slides were then washed in running tap water for 5 minutes, counterstained in Mayer's haematoxylin for 1 minute and washed again for 5 minutes. Sections were dehydrated in gradual IMS washes (70%, 80%, 90% and 100%), incubated twice in xylene for 5 minutes and mounted on the CTM6 Glass Coverslipper (Thermo Fisher Scientific/Microm, Walldorf, Germany). All sections within the same set (i.e., samples A, B and C) were stained in the same batch to minimise any batch-to-batch variations that might occur which could affect staining intensity and lead to erroneous results.

**Table 1:** Details of the immunohistochemical procedure used for each marker*

IHC procedure	Antigen Retrieval	Endogenous Peroxidase Blocking	Monoclonal Antibody	Secondary Antibody	Signal Amplification	Signal Detection	Kit used
p-Erk1/2	Preheated target retrieval solution (1×), water bath 97 ± 2°C, 30 min	Phosphate buffer containing hydrogen peroxide, 5 min	Phospho-p44/42 MAPK (Erk1/2) (Thr202/Tyr204); (1:100) (Cell Signalling, #4376), 1 h	Rabbit linker, 15 min	Polymer conjugated to horseradish peroxidase (HRP) and goat antirabbit and antimouse immunoglobulin, 30 min		EnVision™ FLEX+ (Dako)
p-Akt			Phospho-Akt (Ser473); (1:25) (Cell Signalling, #3787), 1 h				
Ki67	Preheated 0.01 M citrate buffer pH 6.0, microwave 800 W, 10 min		Clone MIB-1 (1:40) (Dako), 20 min	Biotinylated goat antimouse and antirabbit immunoglobulins, 15 min	Streptavidin conjugated to HRP, 15 min	DAB (chromogen) in hydrogen peroxide (substrate), 10 min	Real™ (Dako)
ER			Clone 6F11 (1:40) (Novocastra), 20 min				
PgR			Clone 16 (1:100) (Novocastra), 20 min				
HER2	Preheated 0.01 M citrate buffer, water bath 97 ± 2°C, 40 min	3% hydrogen peroxide, 5 min	Rabbit antihuman HER2 protein, 30 min	Polymer conjugated to HRP and goat antirabbit immunoglobulin, 30 min			HercepTest™ (Dako)

*ER, oestrogen receptor: PgR, progesterone receptor; HER2, human epidermal growth factor receptor 2; HRP, horseradish peroxidase, DAB, 3,3'-diaminobenzidine.

Assessment of p-Akt, p-Erk1/2, ER and PgR expression across 10 high-power fields was done by H score, in which the percentage of invasive cells staining in each intensity category (0, zero; 1, faint; 2, moderate; or 3, strong) was derived, multiplied by its intensity and summed (range, 0-300). Ki67 expression was assessed by percentage of positive invasive cells staining in any category 1-3 (percentage positive score) across 10 high-power fields. HER2 assessment was done by IHC categories 0, 1+, 2+ and 3+ as per HercepTest™ (DakoCytomation, Glostrup, Denmark) and the American Society of Clinical Oncology/College of American Pathologists (ASCO/CAP) guidelines [5]. The assessment of all markers was blinded with regard to sample time point and sample set.

### Statistical analysis

Comparison of IHC scores between samples A, B and C was conducted using the Wilcoxon signed-rank test. Spearman regression analyses were conducted between the difference in expression between samples A and B and the time elapsed between their collection. Data from samples placed in RNAlater will be published separately. *P *values were two-sided.

## Results

Twenty complete sets of samples with adequate invasive tissue and one set missing sample B were available. Median time from collection of samples A and B was 30 minutes (range, 20-80 minutes). No significant differences were found between samples A and B for Ki67, ER, PgR, p-Akt, p-Erk1/2 or HER2 (Table 2). There were no significant correlations between time elapsed and the differences between samples A and B (all *P *> 0.20). Comparisons with sample C were therefore made with mean expression values for samples A and B (mean A,B).

**Table 2:** Median (interquartile range) expression of markers ER, PgR, Ki67, p-Akt and p-Erk1/2 in samples A, B and C as defined in the text*

Marker	Sample A	Sample B	Sample C	*P *value
Ki67	16.4 (9.4-21.7)	17.1 (7.0-25.1)	15.3 (8.0-19.6)	0.19
ER	190.4 (171.2-200.5)	194.7 (167.6-199.4)	188.0 (145.1-195.4)	0.06
PgR	158.9 (58.3-193.3)	161.0 (97.2-189.0)	153.3 (42.2-193.2)	0.89
HER2	3+: 1; 2+: 2; 1+: 14; 0: 4	3+: 1; 2+: 2; 1+: 13; 0: 4	3+: 1; 2+: 2; 1+: 3; 0: 15	0.0012
p-Akt	111.4 (60.8-178.6)	101.1 (75.4-146.0)	26.8 (5.0-75.4)	<0.0001
p-Erk1/2	212.2 (123.6-243.6)	193.1 (113.5-238.0)	69.3 (0.4-113.5)	<0.0001

*Numerical values for samples A, B and C refer to H scores (for ER, PgR, p-Erk1/2 and p-Akt) and percentage positive scores (for Ki67). For HER2, the number of cases in each staining category is shown. *P *values refer to comparison between mean A, B and C samples. ER, oestrogen receptor: PgR, progesterone receptor; HER2, human epidermal growth factor receptor 2.

**Figure 1:**
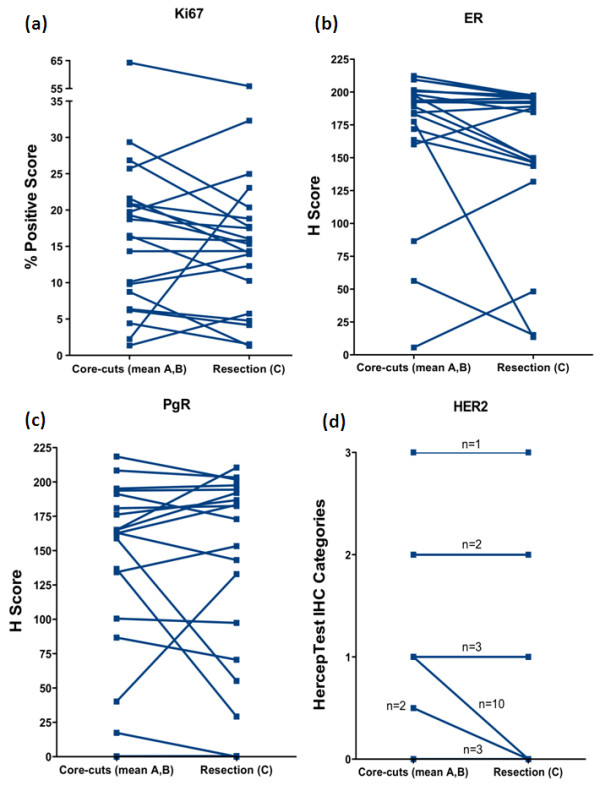
**Expression of commonly assessed markers in breast cancer specimens by immunohistochemistry (IHC)**. Expression of **(a) **Ki67, **(b) **ER, **(c) **PgR and **(d) **HER2 in core-cuts (mean of samples A and B) and resections (sample C).

**Figure 2:**
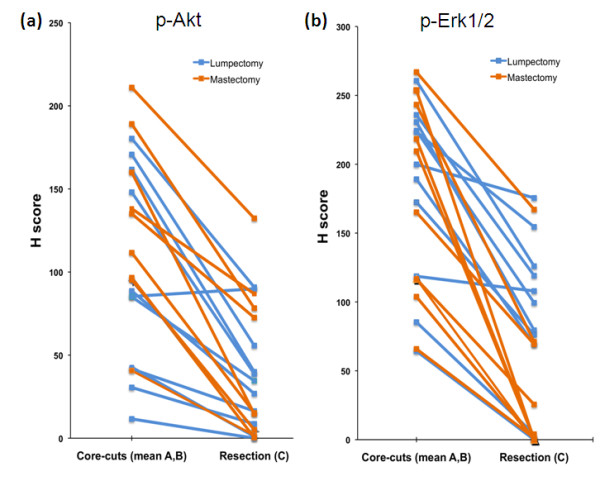
**Expression of phosphoproteins in core-cuts and excisions according to type of surgical specimen**. **(a) **p-Akt and **(b) **p-Erk1/2 expression by IHC in core-cuts (mean of samples A and B) and resection (sample C).

There were no significant differences between these scores for Ki67, ER and PgR, although a trend to a lower ER value in the resection was seen (Figure 1a, *P *= 0.19; Figure 1b, *P *= 0.06; and Figure 1c, *P *= 0.89, respectively). For HER2, 12 of 15 cases categorised as 1+ on at least one of sample A or B were scored 0 in the resection, but no differences in HER2 positive or negative status occurred (Figure 1d). However, p-Akt and p-Erk1/2 were highly significantly lower in sample C than in mean A,B (Figures 2a and 2b, respectively; *P *< 0.0001). Near-complete absence of staining (H score <5) occurred in 6 of 21 samples C for p-Akt and 8 of 21 for p-Erk1/2, despite mean A,B values for some of these being among the highest. Nonetheless, there was a significant correlation between values for mean A,B and C samples for both phosphoproteins (*R *= 0.68, *P *= 0.0008; *R *= 0.52, *P *= 0.015, respectively).

**Figure 3:**
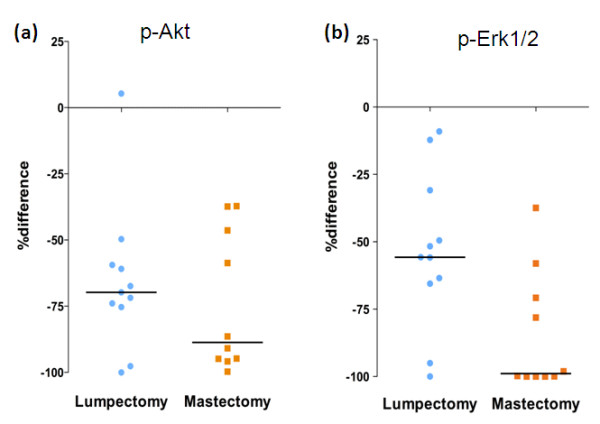
**Expression of p-Akt and p-Erk1/2 in lumpectomy versus mastectomy specimens**. Percentage difference between resections (sample C) and core-cuts (mean of samples A and B) expression values for **(a) **p-Akt and **(b) **p-Erk1/2. Percentage difference defined as (sample C - mean of samples A and B) × 100/mean of samples A and B.

The mean difference in staining between mean A,B and C samples was greater for both phosphoproteins in mastectomy than lumpectomy samples (Figures 3a and 3b), but only significantly so for p-Erk1/2 (*P *= 0.01).

## Discussion

The data described reveal that the immunohistochemical detection of certain biomarkers is highly comparable between core-cut biopsies and routinely fixed resections. The modest difference in ER and the lack of difference in Ki67 and PgR is consistent with the absence of a difference between core-cuts and resection samples in the placebo arm of some of our earlier reports on short-term presurgical studies [6,7]. While there was no effect on the p-Akt or p-Erk1/2 staining over the 20- to 80-minute delay in fixation of core-cuts, the difference between core-cuts and excision samples was extreme and could lead to major erroneous interpretation. For example, if these results were obtained in a "window-of-opportunity" trial that included only a treatment arm, they could be taken as strong evidence of the pharmacological effectiveness of that treatment.

The differences between core-cut and excision samples could be influenced by sampling differences of heterogeneously expressed markers but are most likely due to differences in time to fixation: the correlation between core-cut and excision values indicates a systematic difference. At room temperature and without fixation accelerators, formalin, by far the most widely used histopathological fixative, penetrates tissue at approximately 1 mm/hour [8]. Thus fixation will be initiated rapidly throughout core-cuts (small volume) after immersion whilst penetration of the larger volume main specimen will be slower, resulting in greater loss of biomarker expression. Manoeuvres are therefore necessary to allow rapid initiation of fixation throughout excision biopsies. This is further supported by the reduction in phospho-staining between cores and the main resection being more noticeable in mastectomy than in lumpectomy specimens, especially for p-Erk1/2.

We recently reported that the routine fixation procedure at RMH provides excellent correlations for ER and HER2 status (i.e., positive or negative) between diagnostic core-cuts and resection specimens and those results are supported here [9]. Approximately 15% discordance was noted for PgR in that study on the basis of positive-negative categorisation. Using the cutoff of H score 20, no such discordance was seen in the current study, but the numbers of subjects are too low to exclude that level of discordance. The trend to a quantitative difference reported here for ER and the significant difference we reported previously between excisions and cores are unlikely to lead to erroneous exclusion of responsive patients from endocrine therapy [6]. However, recent data indicate that quantitative levels of ER may be usefully incorporated into algorithms for predicting patient outcome [10]; such assessments will benefit from optimal fixation procedures.

The major differences seen in the two phosphoproteins have major implications for clinical research and future patient management. Past research reports that assessed expression of these markers in samples without special attention to fixation, including studies from our own group [11], may have reached erroneous conclusions [12-19]. One recent publication on p-Akt in relation to the clinical benefit of paclitaxel in the adjuvant treatment of breast cancer recognised the potential for loss of immunoreactivity because of variations in specimen fixation; an optimised antigen retrieval technique was stated to have been used, but its effectiveness was not reported [20]. False-negative results (when inconsistent fixation has occurred) or false-positive results (when systematic bias has resulted from consistently different fixation as alluded to above for window-of-opportunity studies) may both occur. There are also implications for future clinical management: if any such labile markers emerge, as is anticipated, from current translational research efforts, stored routinely fixed tissues are unlikely to be valid for their measurement. Although guidelines for optimal fixation have been published [5], it is clear that these are not uniformly adhered to. This practice needs to be markedly improved to prepare for clinical application of future agents.

## Conclusions

Immunohistochemical staining for ER, PgR, HER2 and Ki67 is similar between core-cuts and excision biopsies but is markedly reduced for p-Akt and p-Erk1/2 in routinely fixed excisions. This effect could lead to both false-negative and false-positive findings according to study design and should be considered both in study design and when interpreting published data.

## Abbreviations

DAB: 3,3"-diaminobenzidine; ER: oestrogen receptor; FFPE: formalin-fixed paraffin-embedded, HER2: human epidermal growth factor receptor 2; HRP: horseradish peroxidase; IHC: immunohistochemistry; IMS: industrial methylated spirits; PGR: progesterone receptor; ASCO: American Society of Clinical Oncology; CAP: College of American Pathologists.

## Competing interests

The authors declare that they have no competing interests.

## Authors' contributions

IP coordinated sample collection, conducted immunohistochemical determination and scoring of markers Ki67, p-Akt and p-Erk1/2, acquired data on clinical-pathological parameters, conducted statistical analysis and interpretation of data for all markers and assisted in drafting the manuscript; FM selected eligible patients, collected samples in theatre and assisted in drafting the manuscript; MH sectioned tissue blocks and conducted immunohistochemical determination and scoring of markers ER, PgR and HER2; SD assisted in the immunohistochemical determination of phospho-markers; RA supervised the statistical analysis and assisted in drafting the manuscript; AN and PO supervised the histopathological processing of samples and contributed towards the histopathological discussions; JS provided training and supervision of the immunohistochemical tests; IS contributed to the design of the study and its interpretation; and MD conceived and designed the study, interpreted data and drafted the manuscript. All authors approved the final manuscript.
